# Synthesis of Ni-Doped Graphene Aerogels for Electrochemical Applications

**DOI:** 10.3390/gels10030180

**Published:** 2024-03-04

**Authors:** Marina González-Barriuso, Mario Sánchez-Suárez, Judith González-Lavín, Ana Arenillas, Natalia Rey-Raap

**Affiliations:** 1 Institute of Carbon Science and Technology (INCAR-CSIC), Calle Francisco Pintado Fe, 26, 33011 Oviedo, Spain; marina.gonzalez@unican.es (M.G.-B.); mario.sanchez@incar.csic.es (M.S.-S.); judith.g.lavin@incar.csic.es (J.G.-L.); aapuente@incar.csic.es (A.A.); 2 Inorganic Chemistry Group, Department of Chemistry and Process and Resource Engineering, School of Industrial and Telecommunication Engineers, University of Cantabria, Avenida de los Castros s.n., 39005 Santander, Spain

**Keywords:** carbon aerogels, graphene, electrical conductivity, porosity, electrochemistry

## Abstract

Carbonaceous materials used in most electrochemical applications require high specific surface area, adequate pore size distribution, and high electrical conductivity to ensure good interaction with the electrolyte and fast electron transport. The development of transition metal doped graphene aerogels is a possible solution, since their structure, morphology, and electrical properties can be controlled during the synthesis process. This work aims to synthesize Ni-doped graphene aerogels to study the role of different nickel salts in the sol-gel reaction and their final properties. The characterization data show that, regardless of the nature of the Ni salts, the surface area, volume of micropores, and enveloped density decrease, while the porosity and electrical conductivity increase. However, differences in morphology, mesopore size distribution, degree of order of the carbon structure, and electrical conductivity were observed depending on the type of Ni salt. It was found that nickel nitrate results in a material with a broader mesopore distribution, higher electrical conductivity, and hence, higher electrochemical surface area, demonstrating that graphene aerogels can be easily synthesized with tailored properties to fit the requirements of specific electrochemical applications.

## 1. Introduction

Electrochemical applications such as supercapacitors, fuel cells, batteries, or sensors require carbon electrode materials that meet certain characteristics, such as the absence of impurities, a high electrical conductivity to ensure fast electron transport, high porosity, and a specific surface area and pore size distribution that are suitable for the application [1,2,3,4]. Specifically, supercapacitors involve carbon electrode materials with high specific surface areas, above 2000 m^2^ g^−1^ [5,6,7,8], combined with a mesoporous structure (the absence of large pores). In fact, the size, distribution, and geometry of the pores have a great influence on the performance of these devices [1,9]. On the other hand, carbon electrodes for fuel cells or sensors must have active centers, such as metal nanoparticles or heteroatoms, and specific surface areas close to 1000 m^2^ g^−1^ [10,11,12], a suitable pore distribution for the transport of reactive gases to the active centers (accessible porosity) [13,14,15]. In the case of batteries, there is not a combination of unique properties, as the properties of the carbon materials used as electrodes depend on the type of battery (lithium-ion, sodium-ion, dual-ion, lithium-air, lithium-sulfur, etc.). For instance, Li-ion batteries require highly structurally ordered materials [16,17]; the anodes for sodium-ion batteries should have small pore volumes to increase their energy density and reduce their reversible capacity [18,19]; in Li–O_2_ batteries, porous materials with large pore volumes and an adjustable pore structure are needed [20]; Li-S batteries involve materials with high specific surface areas [21,22]. Thus, the proper combination of carbon electrode material properties is essential to enhance the performance of these electrochemical applications. However, high porosity and electrical conductivity are antagonistic properties. Electrical conductivity requires ordered and crystalline structures, which implies low-porosity or non-porous structures. Therefore, designing materials that combine these properties is a key challenge in the electrochemical applications field.

The use of synthetic carbon aerogels to solve these problems is postulated as an interesting solution, as their structure, morphology, and electrical properties can be tailored during the synthesis process [23,24,25,26,27]. Carbon aerogels, commonly obtained by resorcinol-formaldehyde (RF) precursor solutions, are porous materials composed of interconnected polymeric clusters. The synthesis of these materials is based on the sol-gel methodology, which allows the final physicochemical properties to be controlled by varying the composition of the precursor solution. The sol-gel reaction also allows for the introduction of heteroatoms or nanostructures into the precursor solution, which can be incorporated into the polymeric network of the aerogel [28,29]. Despite the great advantages of carbon aerogels, they also present some weaknesses, such as their limited electrical conductivity, an essential requirement in electrochemical applications. To address this drawback, graphene aerogels have been synthesized following different strategies [21,30,31,32,33,34,35,36,37,38,39,40,41]. One of these approaches involves the sol-gel reaction, combining graphene structures and RF precursor solutions [31,33,35], resulting in materials with an electrical conductivity ranging from 80 S m^−1^ to 850 S m^−1^ [31,33], with different ranges of porosity, densities, and specific surface areas. Another strategy involves the chemical reduction of a GO suspension, commonly performed by hydrothermal processes [30,32,37,42,43] or by mild reducing agents [42,44,45,46,47], avoiding the use of RF. However, this methodology results in aerogels with lower electrical conductivities and a low mechanical strength. The readers are referred to several interesting reviews on this topic [30,40,41]. Therefore, it is necessary to develop an easy and non-complex method to produce graphene aerogels, combining a high surface area, porosity, and electrical conductivity, while allowing for the design of other properties that are essential to fit the requirements of different electrochemical applications. Some of these properties are related to the incorporation of metals such as iron, nickel, cobalt, titanium, or silicon into the graphene aerogel structure, which has been shown to improve the electrochemical performance of these materials in supercapacitors and batteries [40,48,49]. However, despite this evidence, there are only a few studies in this field, which are focused on the preparation of composites using metal oxides or the addition of chloride salts using complex and time-consuming processes.

Therefore, this work aims to synthesize nickel-doped graphene aerogels in a one-step process with tailored properties, which can then be adapted to design their final properties to fit the requirements of different electrochemical applications. The novelty of this work lies in the study of the effect of the nickel salt employed during the synthesis process on the final properties, which has not yet been reported. The introduction of several nickel salts into the initial precursor solution gives rise to graphene aerogels with different porosities, morphologies, specific surface areas, and degrees of order. Furthermore, the metal nanoparticles have a double function: (i) to increase the electrical conductivity and (ii) to serve as electrochemical active centers. Therefore, this study demonstrates that the developed methodology is an interesting strategy to tailor the final properties of nickel-doped graphene aerogels and fit the requirements of specific electrochemical applications.

## 2. Results and Discussion

**Figure 1:**
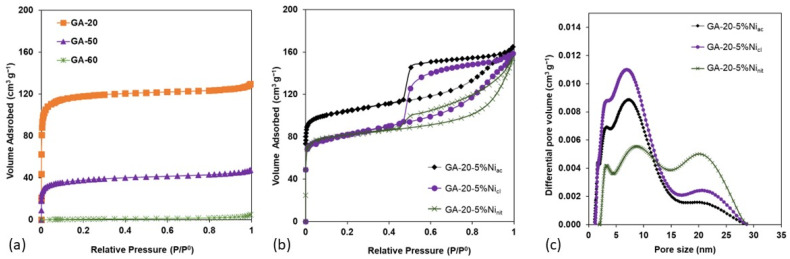
N_2_ adsorption−desorption isotherms of (**a**) graphene aerogels prepared with dilutions of 20, 50, and 60, and (**b**) nickel−doped graphene aerogels with 5% wt. of nickel. (**c**) Pore size distribution (PSD) of nickel-doped graphene aerogels with 5% wt. of nickel.

Graphene aerogels have been synthesized using different dilution ratios, from 20 to 60. The N_2_ adsorption–desorption isotherms are plotted in Figure 1a, while Table 1 shows the values of the specific surface area, enveloped density, porosity, and electrical conductivity of the graphene aerogels.

**Figure 2:**
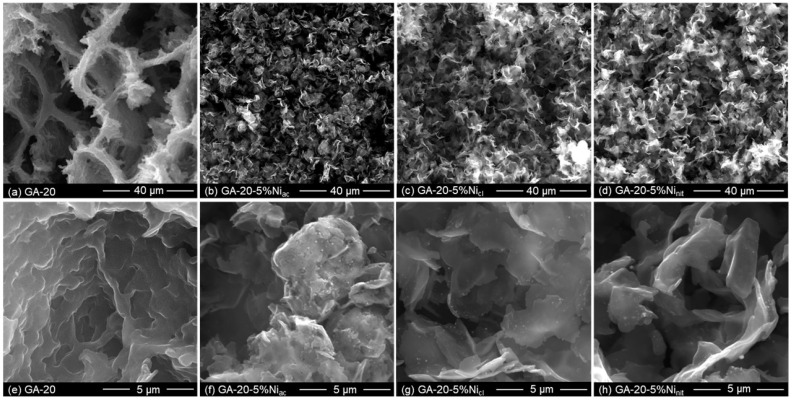
SEM images of (**a**,**e**) undoped graphene aerogels prepared with dilution 20, and nickel-doped graphene aerogels with 5% wt. of nickel using (**b**,**f**) nickel acetate, (**c**,**g**) nickel chloride, and (**d**,**h**) nickel nitrate.

The shape of the isotherm of GA-20 can be ascribed to type I, characteristic of microporous materials (V_micro_ of 0.17 cm^3^ g^−1^). The volume adsorbed at low relative pressures decreases by increasing the dilution ratio due to the different percentages of resorcinol/formaldehyde (R/F) used in the precursor solution, suggesting a decrease in the specific surface area and volume of micropores. Graphene aerogels have been synthesized using a dispersion of graphene oxide, resorcinol, and formaldehyde, in which these last two monomers react to form a microporous carbon gel that acts as a binder to prevent the collapse of the structure and the stacking of the graphene sheets [27], as shown in Figure 2a. Microporosity is generated on the carbon gel produced from R/F, so V_micro_ and the surface area (S_BET_) decrease with the percentage of resorcinol–formaldehyde that is used (from 22% in GA-20 to 8% in GA-60). These results are in agreement with other studies in which GA was synthesized using RF as binder [31,33], and higher than GAs synthesized by hydrothermal processes directly from graphene oxide [42], indicating the importance of adding RF into the precursor solution to avoid the collapse of the structure. On the other hand, the isotherms shown in Figure 1a do not exhibit a hysteresis loop, indicating that these samples do not have mesoporosity (V_meso_ < 0.01 cm^3^ g^−1^, as shown in Table 1).

Even though microporosity can be decreased by increasing the dilution ratio, the total porosity increases, probably due to the larger amount of randomly connected graphene sheets, which leads to a less dense material with a large volume of macropores (which cannot be detected by the N_2_ adsorption–desorption isotherm technique but can be observed by SEM). Figure 2a shows the morphology of GA-20, which is composed of the randomly distributed graphene sheets recovered by the carbon aerogel obtained from RF. Figure 2e shows GA-20 with a higher magnification, in which an interconnected macroporous 3D network structure with wrinkled features of thin graphene sheets can be observed, along with the cover layer of the carbon aerogel obtained from RF. This cellular structure is similar to that observed for other graphene aerogels, obtained from more complex processes involving the use of templates, a long synthesis time, or several steps for solvent exchange [37,42,46,50].

The electrical conductivity increases with the dilution ratio due to an increase in the graphene content, although the increment is not linear, suggesting that the electrical conductivity achieves a maximum value. Based on these results and bearing in mind that this work aims to obtain materials combining a high surface area and electrical conductivity, sample GA-20 was selected for the preparation of nickel-doped graphene aerogels.

The porous properties of Ni-doped graphene aerogels vary with the introduction of nickel salts in the initial precursor solution before the sol-gel synthesis. Regardless of the nature of the Ni salt, the surface area, volume of micropores, and enveloped density decrease, while the porosity and electrical conductivity increase (Table 1). Commonly, the addition of metals into carbon structures gives rise to lower surface areas due to the presence of non-porous metal nanoparticles, decreasing the surface area according to the concentration of the metal. However, data in Table 1 show that the values of S_BET_ decrease by 15% (GA-20-5%Ni_ac_) and 34% (GA-20-5%Ni_cl_ and GA-20-5%Ni_nit_) depending on the Ni salt, which can be also observed in the lower volume adsorbed at a low relative pressure (Figure 1b). Generally, the surface area and microporosity of carbon aerogels can be decreased by increasing the degree of order [17], so this result suggests that the presence of specific Ni salt during the carbonization process performed at 1000 °C favors the ordering of the carbon structure (corroborated by Raman spectroscopy as discussed below). On the other hand, the decrease in the surface area could be also due to the blocking of micropores (pore sizes lower than 2 nm) by metallic particles deposited on the surface. Therefore, the surface area may decrease due to a combination of: (i) the proportion of non-porous metal nanoparticles, (ii) the higher degree of order, and (iii) micropores blocked by the metal nanoparticles, with the last two reasons depending on the nickel salt employed. To shed some light on this discussion, Raman and XRD patterns will later be analyzed.

Another important feature of the N_2_ adsorption–desorption isotherms of Ni-doped graphene aerogels (Figure 1b) is the appearance of a type H4 hysteresis loop at medium–high relative pressures, indicating that the samples generated mesoporosity due to the presence of the nickel salt during the sol-gel reaction. Even though the shape of the mesopores seems to be similar for all the samples according to the type of hysteresis loop, differences can be observed in the volume of mesopores and the pore size distribution (PSD). Sample GA-20-5%Ni_cl_ and GA-20-5%Ni_ac_ exhibit a PSD centered at 7 nm (Figure 1c), being the volume of mesopores higher for GA-20-5%Ni_cl._, while sample GA-20-5%Ni_nit_ also has a lower volume of mesopores but a totally different PSD shape, indicating the presence of mesopores of a larger size. This increase in mesoporosity in comparison to GA-20 also contributes to the increase in the total porosity and, in turn, the lower values of enveloped density.

The introduction of nickel during the synthesis of the graphene aerogels also modifies the characteristic cellular structure, as shown in Figure 2. Metal ions may act as active sites for the assembly of the graphene layers [51], which favors the formation of corrugated layers, giving rise to highly interconnected porous structures that leave smaller voids between the layers, which probably favors the appearance of mesoporosity. This phenomenon shows that nickel plays an important role in the formation of the structure. However, at higher magnifications, some differences are observed due to the nickel salt that is employed (Figure 2f–h). The presence of nickel chloride (Figure 2c,g) and nickel nitrate (Figure 2d,h) during the sol-gel reaction, followed by the carbonization process, results in small flakes that are recovered by the amorphous carbon aerogel obtained from RF, while in the morphology obtained by using nicked acetate (Figure 2b,f), the graphene sheets seem to be rolled on themselves. This could be due to the ordering of the carbon structure during carbonization, especially for those samples doped with nickel chloride and nickel nitrate, in agreement with the results of the BET surface area.

**Figure 3:**
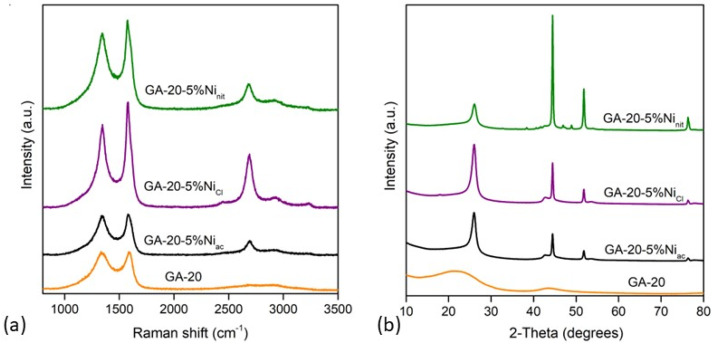
Raman (**a**) and XRD (**b**) patterns of undoped graphene aerogels prepared with dilution 20, and nickel−doped graphene aerogels with 5% wt. of nickel.

The electrical conductivity can be strongly increased by introducing the metal into the aerogel structure (Table 1). However, it increases unevenly depending on the metallic salt used in the synthesis. These differences are initially related to the porosity and degree of order of the carbon structures. The graphitic domains were evaluated by Raman spectroscopy (Figure 3a). The Raman spectra show two main peaks at ca 1350 cm^−1^ and 1580 cm^−1^, attributed to the D and G band, respectively. The D-band is related to disorder or defects in the carbon structure (vacancies or crystal edges), while the G-band is ascribed to the in-plane stretching vibration of the carbon–carbon double bond. The Ni-doped samples also show a peak at around 2690 cm^−1^, which is commonly known as the G′-band or 2D band (second d-order region), and its shape and position depend on the number of graphitic layers. Based on these peaks and the intensity ratio between the D- and G-band (I_D_/I_G_), it can be inferred that the degree of order increases as follows: GA-20 (0.99) < GA-20-5%Ni_ac_ (0.99) < GA-20-5%Ni_nit_ (0.90) < GA-20-5%Ni_cl_ (0.88). GA doped with nickel acetate has a higher specific surface area and a lower degree of order than those synthesized from nickel chloride and nickel nitrate, and thus, a lower electrical conductivity. However, even though its surface area and degree of order are similar to GA-20, the conductivity is 82% higher, suggesting that the presence of nickel nanoparticles and the mesoporosity may also contribute to increases in the *K* value. This statement can be also validated by comparing samples GA-20-5%Ni_cl_ and GA-20-5%Ni_nit_, with a similar surface area, in which GA-20-5%Ni_nit_ exhibits a lower degree of order but a higher electrical conductivity, suggesting once again that *K* also depends on other factors, such as the distribution or crystalline size of nickel nanoparticles.

The structure of the graphene aerogels was further analyzed by X-ray diffraction, and the XRD patterns are shown in Figure 3b. The XRD pattern of the graphene aerogel (GA-20) shows two broad peaks at 2Ɵ values close to 22° and 44°, indicative of an amorphous or turbostratic graphite structure. In contrast, the patterns of Ni-doped samples show a well-defined and sharp diffraction peak at 26°, attributed to graphitic domains, indicating that the structure was graphitized during carbonization due to the presence of nickel. Furthermore, these samples also show three main peaks at 44°, 52°, and 76°, characteristic of face-centered cubic (FCC) crystals of pure nickel nanoparticles (PDF 04-0850) and ascribed to the lattice reflections of (111), (200), and (220), respectively. Therefore, it can be inferred that the nickel salts introduced during the synthesis process were reduced during carbonization, resulting in elemental nickel, which is a phenomenon that is commonly observed in these types of materials [51]. The peaks associated with Ni seem to be more intense in sample GA-20-5%Ni_nit_, suggesting that the degree of crystallinity increases as follows: GA-20-5%Ni_ac_ < GA-20-5%Ni_cl_ < GA-20-5%Ni_nit_. This was further corroborated by the crystal size, calculated according to Scherrer’s equation, obtaining a crystallite size of nickel nanoparticles (determined for the peak detected at 44°) of 24, 26, and 32 nm for GA-20-5%Ni_ac_, GA-20-5%Ni_cl_, and GA-20-5%Ni_nit_, respectively.

**Figure 4:**
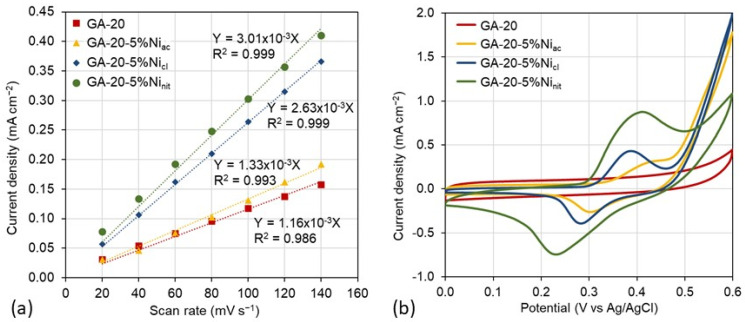
Linear regressions between current density obtained from cyclic voltammograms and scan rates (**a**) and cyclic voltammograms showing the redox reaction of nickel measured at a scan rate of 40 mV s^−1^ (**b**) for undoped graphene aerogels prepared with dilution 20, and nickel−doped graphene aerogels with 5% wt. of nickel.

The ECSA of the samples was evaluated to determine the accessibility to nickel nanoparticles. Figure 4a shows the values of the current density achieved from cyclic voltammograms recorded in a non-faradaic region versus the scan rates (CVs can be found in Figure S1 of Supplementary Materials). The slope of the regression could be related to the ECSA values, as explained in the experimental section. It can be observed that sample GA-20-5%Ni_ac_ has a similar value to GA-20, in agreement with the physicochemical characterization, as both samples exhibit a similar surface area, electrical conductivity, and degree of graphitization. The ECSA value doubles for GA-20-5%Ni_cl_, and increases even further for GA-20-5%Ni_nit_, probably due to the morphology of these structures, which favors accessibility to the nickel nanoparticles, especially the larger mesopores present in GA-20-5%Ni_nit_ and the higher degree of crystallization. This latter fact is of great relevance in electrochemical applications, so it was also corroborated by performing CV at 40 mV s^−1^, within the potential range of the redox reactions of nickel (Figure 4b). As expected, sample GA-20 does not exhibit any hump, while the CVs of Ni-doped GA samples present a one-pair, well-separated redox peak. Sample GA-20-5%Ni_nit_ exhibited the most intense humps close to 0.4 V (oxidation) and 0.25 V (reduction), attributed to the reversible transformation of nickel through the electrosorption of OH- ions (β-Ni(OH)2/β-NiOOH couple) [52]. The intensity of the peaks decreases as follows: GA-20-5%Ni_nit_ > GA-20-5%Ni_cl_ > GA-20-5%Ni_ac_, which is in agreement with the ECSA values.

## 3. Conclusions

Graphene aerogels were synthesized using different dilution ratios, which are directly related to the amount of graphene oxide that is employed. It has been observed that the electrical conductivity and porosity increase with the dilution ratio, while the specific surface area decreases. This is due to the increase in the amount of graphene oxide (which provides electrical conductivity) in relation to the amount of resorcinol–formaldehyde (which provides microporosity) in the precursor solution. On the other hand, the incorporation of nickel salts during the synthesis produces Ni-doped graphene aerogels with lower surface area, volume of micropores, and enveloped density, but a higher porosity and electrical conductivity. These modifications in the physicochemical properties are due to the presence of the nickel salts that favor the formation of mesopores inside the structure during the sol-gel reaction and the ordering of the carbon structure during the carbonization process. However, differences are observed due to the nature of the metallic salt. The graphene aerogel doped with nickel nitrate exhibits the highest electrical conductivity (2772 S m^−1^) and a broader mesopore size distribution, which favors the accessibility to the nickel nanoparticles and, in turn, its electrochemical surface area, which is of paramount importance for electrochemical applications. Therefore, Ni-doped graphene aerogels that combine a high porosity, electrical conductivity, and electroactive nickel nanoparticles can be easily prepared via a simple and effective methodology, resulting in materials that can be synthesized according to the needs of the target application. Notwithstanding, it should be considered that, in this study, three different nickel salts have been added to the precursor solution of graphene aerogels to obtain a final percentage of nickel that is close to 5wt.%, so the conclusions are based on these conditions. Further research to elucidate the effect of adding a higher or lower percentage of nickel salt could allow for a more precise design for these materials. On the other hand, the electroactivity of these samples should be compared with the Ni-doped graphene aerogels obtained by the incipient wetness impregnation to demonstrate the effectiveness of the methodology developed in this study. Finally, in future works, this methodology will be applied to design similar materials that will be evaluated in real electrochemical applications, such as fuel cells, electrolyzers, and electrochemical sensors.

## 4. Materials and Methods

Graphene aerogel (GA) was prepared by resorcinol–formaldehyde (RF) polycondensation reactions using a suspension of graphene oxide as the solvent. The preparation of the precursor solution was carried out using the following reagents: 5 mg mL^−1^ graphene oxide (GO) aqueous suspension (ApplyNano Solutions S.L., Alicante, Spain), 37 wt.% formaldehyde solution (F, Sigma-Aldrich, Madrid, Spain), and resorcinol (R, Sumitomo Chemical Co Ltd., Tokyo, Japan). GA was prepared by dissolving the resorcinol in the aqueous suspension of GO by magnetic stirring for 45 min. Once dissolved, formaldehyde was added. The pH was then adjusted to 5 by adding the required amount of a NaOH aqueous solution. The sol-gel and curing stages were carried out in an oven at 85 °C in a glass container sealed with parafilm. The drying stage was carried out by sublimation in a HyperCOOL HC3110 freeze-dryer (Gyrozen Co., Ltd., Kimpo, Republic of Korea) for 48 h. Once dried, the material was carbonized at 1000 °C for 1h under a nitrogen atmosphere. Three samples were prepared following this methodology but with different dilution ratios: 20, 50, and 60. The dilution ratio is defined as the molar ratio between the total solvent and reactants. Total solvent refers to the water and methanol contained in the formaldehyde and the water from the GO suspension, whilst reactants refer to the resorcinol and formaldehyde. Table 2 shows the quantities, in grams, of each reagent used in the preparation of the graphene aerogels. The samples were labeled as GA, followed by the value of the dilution ratio that was employed. For example, GA-20 corresponds to a graphene aerogel synthesized from a dilution ratio of 20. Nickel-doped graphene aerogels were prepared analogously to GA-20 except that, during the dissolution of the resorcinol in the GO suspension, the chosen metal salt was also dissolved. The metal salt was added in the necessary proportion to obtain graphene aerogels with 5 wt.% of nickel (the added quantities are detailed in Table 2). The doped samples were labeled as GA-20-5%Ni_X_, where *X* stands for each type of nickel salt employed: *ac* for nickel (II) acetate tetrahydrate (Alfa-Aesar, Haverhill, MA, USA), *cl* for nickel (II) chloride hexahydrate (Sigma-Aldrich), and *nit* for nickel (II) nitrate hexahydrate (Alfa-Aesar).

The textural properties of the materials were characterized by measuring the envelope density (ρ_env_), and total porosity (ε) in a GeoPyc 1365, the He density in an AccuPyc II 1345, and the specific surface area (S_BET_), the volume of micropores (V_micro_), the volume of mesopores (V_meso_), and the pore size distribution (PSD) in a TriStar II by performing N_2_ adsorption/desorption isotherms at −196 °C (all equipment from Micromeritics, Norcross, GA, USA). All the materials were degassed at 120 °C overnight before the characterization. The electrical conductivity (*K*) measurements were performed using the four-probe method (Everbeing SR-4-6L, Hsinchu, Taiwan) applied to disc-shaped pellets, prepared by mixing 90 wt.% of each GA and 10 wt.% of polytetrafluoroethylene (PTFE by Sigma-Aldrich) in absolute ethanol. Once a homogeneous paste was obtained, it was rolled out to form a thin film from which the pellets were punched. The punched pellets were pressed at 10 t for 30 s to ensure a packed density and then dried at 60 °C overnight. The dimensions of the pellets were 1 cm in diameter and 0.2 mm in thickness. The morphology of the graphene aerogels was examined by a Quanta FEG 650 (FEI Company, Hillsboro, OR, USA) scanning electron microscope (SEM) equipped with an Everhart–Thornley detector. A Raman Renishaw InVia Qontor spectrometer (Wotton-under-Edge, UK) was employed to measure the Raman spectra with a 532 nm laser and 1800 lines mm^−1^ grating with an exposure time of 30 s. The X-ray diffraction patterns were recorded in a D8 Advance diffractometer from Bruker (Billerica, MA, USA) equipped with a CuK_α_ X-ray source. The scans were performed from 5 to 90° with a 0.02° step and 3 s of acquisition time. The electrochemically active surface area (*ECSA*) is commonly calculated by dividing the double-layer capacitance (*Cdl*) by the specific capacitance of a reference material (*Cs*) [53,54], as shown in the SI. All samples of this study are of the same nature, so *Cs* should have the same value for all GA, meaning that the trend of the *Cdl* values could be equivalent to that of ECSA. *Cdl* was determined by performing cyclic voltammetry (CV) in a three-electrode cell involving the GA samples, an Ag/AgCl electrode, and a carbon electrode as the working, reference, and counter-electrodes, respectively. The electrodes were immersed in a 0.1 M KOH solution and the CVs were recorded between 0 and 0.3 V (vs. Ag/AgCl), in which non-Faradic processes take place, at different scan rates (from 20 to 140 mV s^−1^). Then, *Cdl* was calculated as the slope obtained by plotting the maximum current density (at a potential of 0.15 V) versus the scan rate. In addition, CVs were also recorded in a wider potential window, ranging from 0 to 0.6 V (vs. Ag/AgCl), to evaluate the redox reaction of nickel nanoparticles.

## Figures and Tables

**Table 1:** Textural properties and electrical conductivity of undoped and Ni-doped graphene aerogels.

Material	S_BET_ (m^2^ g^−1^)	V_micro_ (cm^3^ g^−1^)	V_meso_ (cm^3^ g^−1^)	ρ_env_ (g cm^−3^)	ε (%)	*K* (S m^−1^)
GA-20	480	0.17	0.01	0.24	87.8	231
GA-50	143	0.05	0.01	0.14	91.4	1290
GA-60	5	- ^a^	- ^a^	0.12	91.6	1447
GA-20-5%Ni_ac_	408	0.14	0.10	0.11	95.1	1303
GA-20-5%Ni_cl_	315	0.11	0.13	0.16	92.4	2390
GA-20-5%Ni_nit_	317	0.11	0.10	0.16	93.6	2772

^a^ values lower than 0.01 cm^3^ g^−1.^

**Table 2:** List of quantities of each reagent required for the preparation of the graphene aerogels (GA) synthesized for this work.

Material	R (g)	GO Suspension (g)	F (g)	C_4_H_6_NiO_4_ (g)	NiCl_2_ (g)	Ni(NO_3_)_2_ (g)
GA-20	4.5	40.4	6.7	-	-	-
GA-50	1.9	45.3	2.8	-	-	-
GA-60	1.6	46.0	2.4	-	-	-
GA-20-5%Ni_ac_	4.5	40.4	6.7	0.4	-	-
GA-20-5%Ni_cl_	4.5	40.4	6.7	-	0.5	-
GA-20-5%Ni_nit_	4.5	40.4	6.7	-	-	3.3

## Data Availability

Data needed to evaluate the conclusions in the paper are present in the manuscript. Additional raw/processed data related to this study may be requested from the authors.

## Supplementary Materials

The following supporting information can be downloaded at: https://www.mdpi.com/article/10.3390/gels10030180/s1, Figure S1. Cyclic voltammograms recorded between 0 and 0.3 V (vs Ag/AgCl), in which non-Faradic processes take place, at different scan rates (from 20 to 140 mV s^−1^) for sample GA-20 (a), GA-20-5%Ni_ac_ (b), GA-20-5%Ni_Cl_ (c), and GA-20-5%Ni_nit_ (d).
