# Supplementary material for: Synthesis of Ni-Doped Graphene Aerogels for Electrochemical Applications
Source: Gels. 2024 Mar 4;10(3):180. doi: 10.3390/gels10030180 (PMC10970258; doi:10.3390/gels10030180)
Supplement: Supplementary file 1 [file gels-10-00180-s001.zip › gels-2876112-supplementary.pdf]

gels

Supplementary Data

Synthesis of Ni-Doped Graphene Aerogels for Electrochemical Applications

Marina González-Barriuso [1,2], Mario Sánchez-Suárez [1], Judith González-Lavín [1], Ana Arenillas [1] and Natalia Rey-Raap [1,*]

1 Institute of Carbon Science and Technology (INCAR-CSIC), Calle Francisco Pintado Fe, 26, 33011 Oviedo, Spain; marina.gonzalez@unican.es (M.G.-B.); mario.sanchez@incar.csic.es (M.S.-S.); judith.g.lavin@incar.csic.es (J.G.-L.); aapuente@incar.csic.es (A.A.)
2 Inorganic Chemistry Group, Department of Chemistry and Process and Resource Engineering, School of Industrial and Telecommunication Engineers, University of Cantabria, Avenida de los Castros s.n., 39005 Santander, Spain
* Correspondence: natalia.rey@incar.csic.es

Electrochemically Active Surface Area (ECSA)

The electrochemically active surface area (*ECSA*) is commonly calculated by the following equation:

$$ECSA = Cdl/Cs$$

where *Cdl is* the double-layer capacitance and *Cs* the specific capacitance of a reference material (*Cs*). It is quite complex to know or determine the exact value of *Cs*, especially for inorganic/organic hybrid materials or material with complex structures, so most of the reported publications assumed that *Cs* have the same value for all the studied materials or at least range around the same order of magnitude. Thus, the trend of the *Cdl* values can be equivalent to that of ECSA. *Cdl* was determined by performing cyclic voltammetry (CV) in a three-electrode cell involving the studied samples, an Ag/AgCl electrode, and a glassy carbon electrode as the working, reference, and counter electrodes, respectively. The electrodes were immersed in a 0.1 NaOH solution and the CV were recorded between 0 and 0.3 V (vs Ag/AgCl), in which non-Faradaic processes take place, at different scan rates (from 20 to 140 mV s^{-1}). Then, *Cdl* was calculated as the slope obtained by plotting the maximum current density (at a potential of 0.15 V) versus the scan rate. Figure S1 shows the CV measured at different scan rates for the undoped sample GA-20 and Ni-doped graphene aerogels.

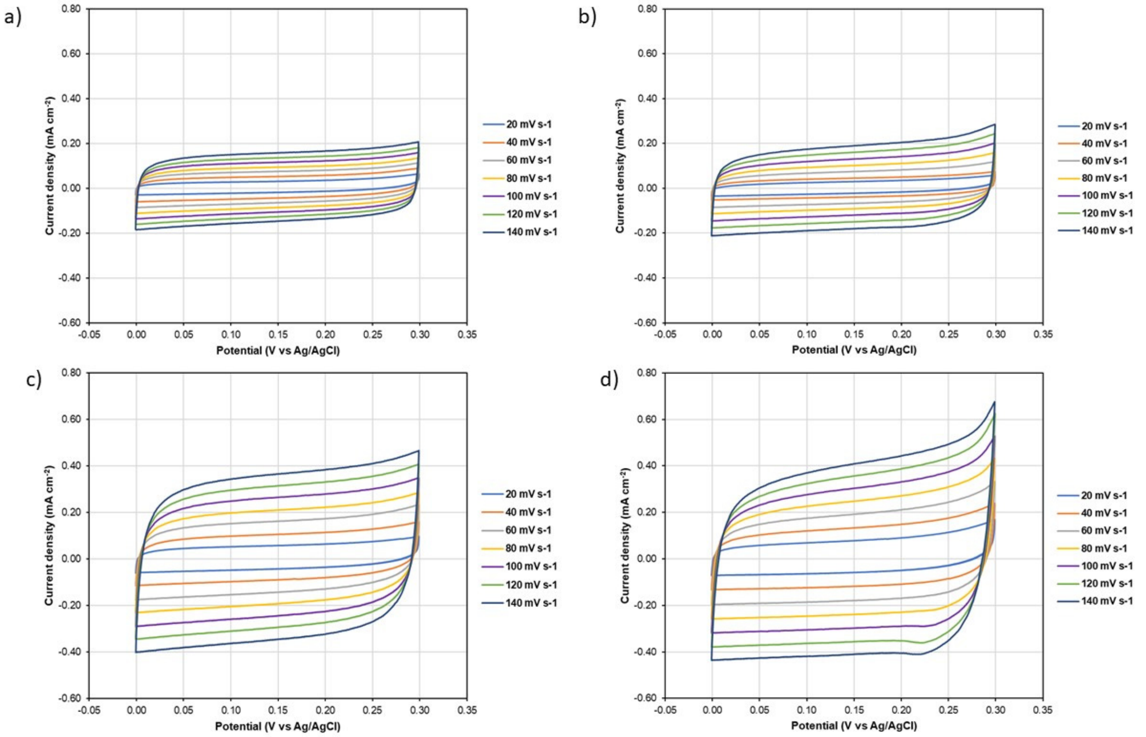

Figure S1. Cyclic voltammograms recorded between 0 and 0.3 V (vs Ag/AgCl), in which non-Faradaic processes take place, at different scan rates (from 20 to 140 mV s^{-1}) for sample GA-20 (a), GA-20-5%Ni_{ac} (b), GA-20-5%Ni_{Cl} (c), and GA-20-5%Ni_{nit} (d).
